# Predictive value of NLR and PLR in response to preoperative chemotherapy and prognosis in locally advanced gastric cancer

**DOI:** 10.3389/fonc.2022.936206

**Published:** 2022-08-30

**Authors:** Wentao Wang, Yilin Tong, Shulan Sun, Yuen Tan, Zexing Shan, Fan Sun, Chengyao Jiang, Yanmei Zhu, Jianjun Zhang

**Affiliations:** ^1^ Department of Gastric Surgery, Liaoning Cancer Hospital and Institute, Cancer Hospital of China Medical University, Shenyang, China; ^2^ Department of Central Laboratory, Liaoning Cancer Hospital and Institute, Cancer Hospital of China Medical University, Shenyang, China; ^3^ Department of Pathology, Liaoning Cancer Hospital and Institute, Cancer Hospital of China Medical University, Shenyang, China

**Keywords:** neutrophil-to-lymphocyte ratio, platelet-to-lymphocyte ratio, gastric cancer, tumor regression grade, prognosis

## Abstract

**Purpose:**

Pretreatment neutrophil-to-lymphocyte (NLR) and platelet-to-lymphocyte (PLR) ratios are markers of systemic inflammation. In patients with locally advanced gastric cancer (GC), the utility of these ratios in predicting tumor regression grade (TRG) after neoadjuvant chemotherapy (NCT) remains unclear.

**Methods:**

This retrospective study examined 283 locally advanced GC patients who underwent NCT and radical surgery. The receiver operating characteristic (ROC) curve analysis and the Youden index were applied to identify optimal NLR/PLR cutpoints. The Kaplan–Meier method was used to estimate overall survival (OS) and disease-free survival (DFS). Univariate/multivariate analyses were conducted by the logistic regression method.

**Results:**

TRG grade proved significantly worse in patients with high values of both NLR and PLR whether in univariate (OR = 3.457; *p* = 0.044) or multivariate (OR = 6.876; *p* = 0.028) analysis. The degree of tumor differentiation was an independent predictive factor for TRG (OR = 2.874; *p* = 0.037) in multivariate analysis. In the subgroup analyses, NLR predicted OS (*p* = 0.04) and DFS (*p* = 0.03) in female patients, whereas PLR was predictive of both OS (*p* = 0.026) and DFS (*p* = 0.018) in patients with clinical TNM stage 3 disease and dissected lymph node counts <28. PLR similarly predicted OS in patients <65 years old (*p* = 0.049), those with positive lymph nodes (*p* = 0.021), or those with moderate or poorly differentiated tumors (*p* = 0.049).

**Conclusion:**

Pretreatment NLR and PLR together serve to independently predict TRG after NCT and surgery in patients with locally advanced GC. Screening for patients with high NLR and PLR values may allow them to benefit upfront from alternatives to NCT.

## Background

Gastric cancer (GC) is currently the fifth most common cancer worldwide and ranks fourth in mortality among tumors globally (1). Pertinent treatment guidelines recommend neoadjuvant chemotherapy (NCT) for patients with locally advanced gastric cancer (2), given its capacity to restrict micrometastases, shrink tumor volumes, boost R0 resection rates, and improve prognoses (3–6).

Although preoperative NCT administration has fueled much progress in recent years, the overall remission rate of chemotherapy is still <50% (7, 8). Because the efficacy of NCT is typically gauged through tumor regression grading (3, 6), based on postoperative pathology assessments, it would be best to identify preoperative markers of NCT efficacy, selecting those patients who may benefit the most.

Inflammation is considered an important marker of cancer growth and progression. Many studies have in fact characterized cancer-associated inflammation as an immune interface phenomenon. Such inflammatory influx often precedes tumor onset and promotes its subsequent development (9, 10). Neutrophil levels may be used independently or as part of the neutrophil-to-lymphocyte ratio (NLR) to predict tumorigenesis and prognosis (11). Some studies have proven that high NLR could predict poor survival in advanced colorectal cancer and malignant pleural mesothelioma (12, 13). Platelet counts and platelet-to-lymphocyte ratios (PLRs) are also cancer-related inflammatory markers (14–18). PLR is a potential prognosticator in patients with pancreatic cancer and is known to predict poor prognosis in patients with colorectal cancer (18). The merits of NLR and PLR as prognostic tools have been shown in several studies assessing non-neoadjuvant chemotherapies for various cancers (15–17). However, few researchers have explored the relations between these ratios and the chemotherapeutic efficacy or prognosis in recipients of NCT.

To address this issue, we evaluated whether NLR and PLR correlate with clinicopathological factors, TRG, or patient prognosis after NCT and surgical resection of locally advanced GC.

## Material and methods

### Patients

We collected 3,196 patient records related to GC surgery in Liaoning Cancer Hospital and Institute from January 2010 to July 2016. The inclusion criteria were 1) patients with pathologically confirmed gastric adenocarcinoma, 2) patients with locally advanced GC [clinical stages II–III of the eighth American Joint Committee on Cancer (AJCC)], 3) patients who underwent radical gastrectomy, and 4) patients who received NCT with or without postoperative treatment. The exclusion criteria were 1) patients who had undergone radiotherapy before surgery; (2) patients diagnosed with residual GC or other malignancies; 3) patients who had diseases that affect the NLR and PLR, such as infections and blood disorders; and 4) patients with incomplete clinicopathological data. Two hundred and ninety cases were locally advanced GC patients who received neoadjuvant therapy, and 7 cases were excluded because of incomplete data. Finally, 283 cases were included in this research. The study was approved by the Ethics Committee of Liaoning Cancer Hospital and Institute.

### Pathological response assessment

The relative slices or blocks were obtained from our biospecimen library. All pathological slices were examined separately by two experienced gastrointestinal pathologists without knowledge of clinicopathological information. Pathological TNM stage after chemotherapy (ypTNM) was re-evaluated according to the AJCC cancer staging guidelines. According to the College of American Pathologists (CAP) system, the pathological response of all primary tumors was analyzed: TRG 0 (no remaining cancer cells, i.e., complete response), TRG 1 (small groups of cancer cells or single cancer cells, i.e., near complete response), TRG 2 (more residual tumors with significant tumor regression, i.e., partial response), and TRG 3 (a significant amount of residual cancer with no evidence of tumor regression, i.e., poor or no response). The consensus between pathologists was reached by using a multiheaded discussion, when there was disagreement. During the evaluation process, other clinicopathological features were reconfirmed. In this study, TRG 0–1 was classified as a response group and TRG 2–3 was classified as a non-response group.

### Blood indicators and data collection

Data on clinical and pathological characteristics of all patients were collected retrospectively and formed an anonymous, proprietary database. All relative data before treatment were extracted from our database, including the counts of neutrophils, lymphocytes, and platelets. The definition of NLR was the absolute value of neutrophil count (ANC) divided by the absolute value of lymphocyte count (ALC) (NLR = ANC/ALC), and PLR was the absolute value of platelet count (PLT) divided by ALC (PLR = PLT/ALC). All hematological tests were conducted in our center according to standardized operating procedures, with consistent testing instruments, reference intervals, and good data consistency.

### Statistical methods

The optimal cutoff value was calculated from the ROC curves and the Youden index. The chi-square test was used to calculate the relationship between categorical variables. The Kaplan–Meier method was used to determine overall survival (OS) and disease-free survival (DFS), and survival differences were compared using the log-rank test. The correlation between clinicopathological factors and TRG was assessed using logistic regression models, and factors with a *p-*value ≤0.05 were included in the multivariate analyses. OS was calculated based on the date of diagnosis and the date of death or last follow-up, whereas DFS was the time from surgery to the date of recurrence or the date of last follow-up. SPSS 25.0 (IBM Corp., Armonk, NY, USA) software was used to analyze the data.

## Results

### Patient characteristics

The baseline patient characteristics are summarized in **Table 1**. Patient age ranged from 25 to 77 years (median, 59 years). There were 211 men (74.6%) and 72 women (25.4%). Tumor locations were distributed as follows: lower third, 168 (59.4%); upper third and gastroesophageal junction (UGEJ), 38 (13.4%); and middle third, 53 (18.7%). Overall, there were 24 (8.5%) diffuse gastric carcinomas (Borrmann type IV). Tumor size was <5 cm in 111 patients (39.2%) and ≥5 cm in 172 patients (60.8%). In lymph node status, 99 (35.0%) were negative and 184 (65.0%) were positive. There were 166 (58.7%) and 117 (41.3%) patients whose dissected lymph node counts were <28 and ≥28, respectively. Histologically, all tumors were adenocarcinomas: 68 (24.0%) well differentiated and 215 (76.0%) moderately or poorly differentiated. According to Lauren’s criteria, 138 (48.8%) were intestinal type, and 145 (51.2%) were diffuse or mixed type. The predominant chemotherapies administered were first-line regimens, such as S-1 plus oxaliplatin (SOX) or oxaliplatin and capecitabine (XELOX) with 2–4 cycles before surgery. At least 2 cycles of postoperative chemotherapy are counted as received postoperative chemotherapy.

**Table 1:** Association of baseline characteristics to CAP-TRG.

Variable	*n* (%) (*n* = 283)	TRG 0–1 (%) (*n* = 88)	TRG 2–3 (%) (*n* = 195)	*p*-value
Age				0.731
<65	215 (76.0)	68 (31.6)	147 (68.4)	
≥65	68 (24.0)	20 (29.4)	48 (70.6)	
Gender				0.857
Male	211 (74.6)	65 (30.8)	146 (74.6)	
Female	72 (25.4)	23 (31.9)	49 (68.1)	
cTNM				0.010
2	95 (33.6)	39 (41.1)	56 (58.9)	
3	188 (66.4)	49 (26.1)	139 (73.9)	
Location				0.064
UGEJ	38 (13.4)	9 (23.7)	29 (76.3)	
Middle third	53 (18.7)	24 (45.3)	29 (54.7)	
Lower third	168 (59.4)	50 (29.8)	118 (70.2)	
Diffuse	24 (8.5)	5 (20.8)	19 (79.2)	
Tumor size				<0.001
<5 cm	111 (39.2)	53 (47.7)	58 (52.3)	
≥5 cm	172 (60.8)	35 (20.3)	137 (79.7)	
Lymph node status				<0.001
Negative	99 (35.0)	53 (53.5)	46 (46.5)	
Positive	184 (65.0)	35 (19.0)	149 (81.0)	
Dissected lymph node counts				0.719
<28	166 (58.7)	53 (31.9)	113 (68.1)	
≥28	117 (41.3)	35 (29.9)	82 (70.1)	
Nervous invasion				<0.001
No	215 (76.0)	81 (37.7)	134 (62.3)	
Yes	68 (24.0)	7 (10.3)	61 (89.7)	
Lymphovascular invasion				<0.001
No	211 (74.6)	78 (37.0)	133 (63.0)	
Yes	72 (25.4)	10 (13.9)	62 (86.1)	
Histological type				0.001
Adenocarcinoma	181 (64.0)	69 (38.1)	112 (61.9)	
Poorly cohesive carcinoma	102 (36.0)	19 (18.6)	83 (81.4)	
Grade				<0.001
Well	68 (24.0)	43 (63.2)	25 (36.8)	
Moderate or poor	215 (76.0)	45 (20.9)	170 (79.1)	
Lauren classification				0.002
Intestinal	138 (48.8)	55 (39.9)	83 (60.1)	
Diffuse or mixed	145 (51.2)	33 (22.8)	112 (77.2)	
ypT				<0.001
0–2	64 (22.6)	55 (85.9)	9 (14.1)	
3–4	219 (77.4)	33 (15.1)	186 (84.9)	
ypN				<0.001
0	99 (35.0)	53 (53.5)	46 (46.5)	
1	47 (16.6)	18 (38.3)	29 (61.7)	
2	75 (26.5)	15 (20.0)	60 (80.0)	
3	62 (21.9)	2 (3.2)	60 (96.8)	
ypTNM				<0.001
1	50 (17.7)	45 (90.0)	5 (10.0)	
2	71 (25.1)	26 (36.6)	45 (63.4)	
3	162 (57.2)	17 (10.5)	145 (89.5)	
Postoperative chemotherapy				0.677
No	29 (10.2)	10 (34.5)	19 (65.5)	
Yes	254 (89.8)	78 (30.7)	176 (69.3)	

TRG, tumor regression grade; UGEJ, upper third and gastroesophageal junction; cTNM, clinical TNM stage; ypTNM, post-neoadjuvant pathologic stage.

### Pathological response in relation to clinicopathological factors and prognosis

**Figure 1:**
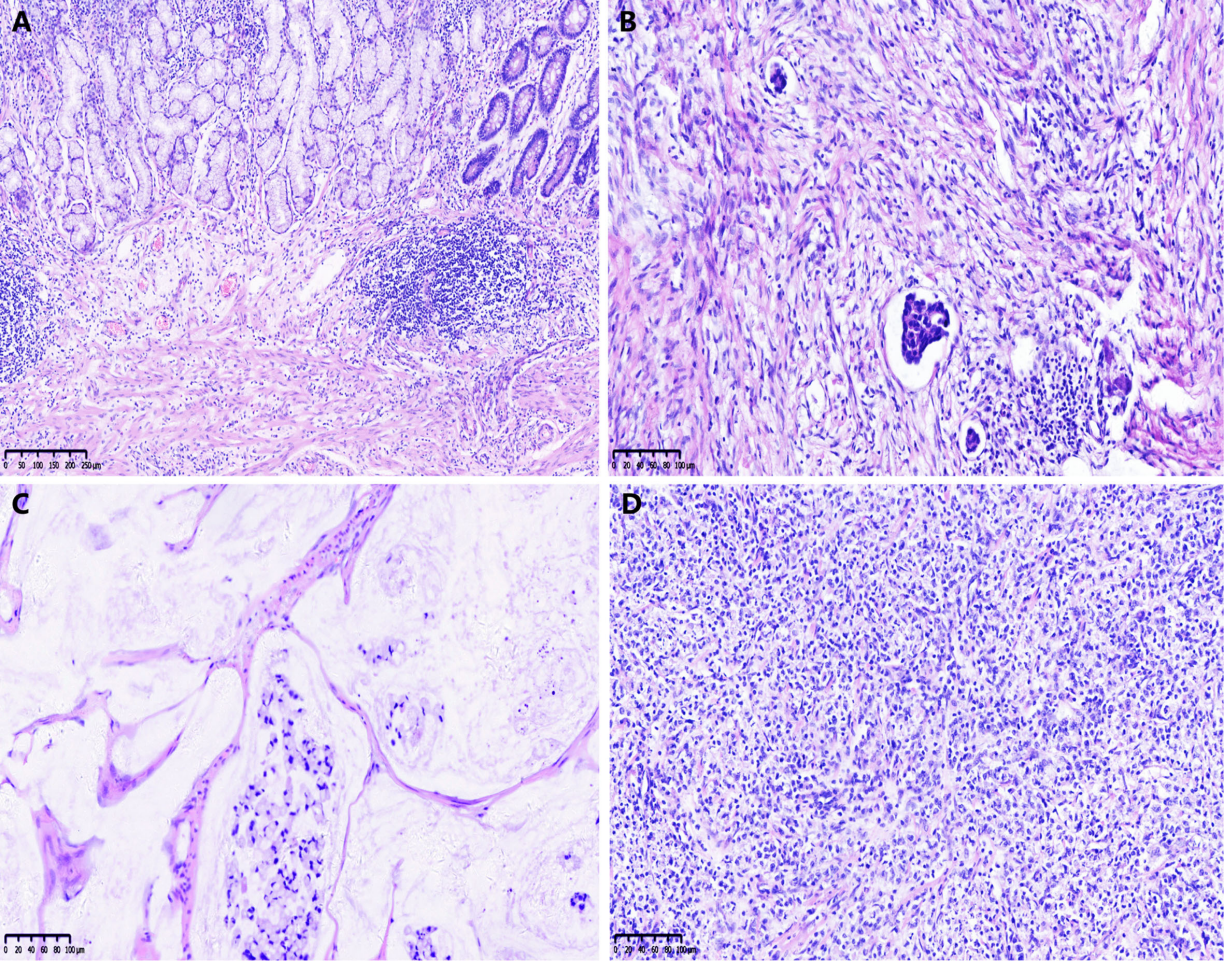
Examples of the College of American Pathologists tumor regression grade (CAP-TRG) system. **(A)** TRG 0, complete tumor regression; **(B)** TRG 1, rare residual tumor; **(C)** TRG 2, significant tumor regression; **(D)** TRG 3, residual tumor without regression.

The CAP-TRG system was used to classify post-NCT responses. Representative images are shown in **Figure 1**.** **A total of 1,280 pathological slices were re-evaluated. After reassessment, there were 8, 80, 65, and 130 patients for the TRG 0–3 subsets, respectively. Good tumor regression (TRG 0–1) was shown by 88 patients (31.1%), and 195 patients (68.9%) displayed poor tumor regression (TRG 2–3).

In **Table 1**, the clinicopathological features are listed according to CAP-TRG class. Clinical TNM stage 3 disease (*p* = 0.010), tumor size ≥5 cm (*p* < 0.001), positive lymph nodes (*p* < 0.001), nervous invasion (*p* < 0.001), lymphovascular invasion (*p* < 0.001), poorly cohesive carcinoma (*p* = 0.001), moderate or poor tumor differentiation (*p* < 0.001), diffuse or mixed Lauren classification (*p* = 0.002), and ypTNM stage 2–3 disease (*p* < 0.001) were all associated with CAP-TRG 2–3, i.e., poorer chemotherapeutic response.

**Figure 2:**
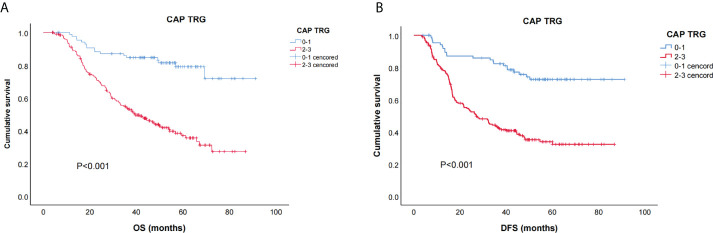
Kaplan–Meier curves for overall survival (OS) **(A)** and disease free survival (DFS) **(B)** of CAP-TRG.

The survival curves for TRG are shown in **Figure 2**: the TRG 0–1 group fared significantly better than the TRG 2–3 group for OS and DFS (both *p* < 0.01). 

### Relations between NLR, PLR, and baseline characteristics

**Table 2** shows the relations between NLR, PLR, and clinicopathological characteristics. The calculated cutpoints were based on the ROC curve and the Youden index, defining high NLR as ≥2.38 and high PLR as ≥188.1. There were 183 patients (64.7%) qualifying as low NLR, 100 (35.3%) as high NLR, 196 (69.3%) as low PLR, and 87 (30.7%) as high PLR. Among all factors, NLR showed significant associations with patient TRG (*p* = 0.015) and nervous invasion (*p* = 0.009), whereas PLR was associated with TRG (*p* = 0.05) and tumor size (*p* = 0.001). The higher the NLR and PLR proved, the worse the pathological response.

**Table 2:** Association of baseline characteristics to NLR or PLR.

Variable	*n* (%) (*n* = 283)	NLR			PLR		
		Low (%) (*n* = 183)	High (%) (*n* = 100)	*p*-value	Low (%) (*n* = 196)	High (%) (*n* = 87)	*p*-value
Age				0.765			0.381
<65	215 (76.0)	138 (64.2)	77 (35.8)		146 (67.9)	69 (32.1)	
≥65	68 (24.0)	45 (66.2)	23 (33.8)		50 (73.5)	18 (26.5)	
Gender				0.681			0.150
Male	211 (74.6)	135 (64.0)	76 (36.0)		151 (71.6)	60 (28.4)	
Female	72 (25.4)	48 (66.7)	24 (33.3)		45 (62.5)	27 (37.5)	
cTNM				0.499			0.446
2	95 (33.6)	64 (67.4)	31 (32.6)		63 (66.3)	32 (32.7)	
3	188 (66.4)	119 (63.3)	69 (36.7)		133 (70.7)	55 (29.3)	
Location				0.929			0.391
UGEJ	38 (13.4)	24 (63.2)	14 (36.8)		29 (76.3)	9 (23.7)	
Middle third	53 (18.7)	34 (64.2)	19 (35.8)		40 (75.5)	13 (24.5)	
Lower third	168 (59.4)	108 (64.3)	60 (35.7)		110 (65.5)	58 (24.5)	
Diffuse	24 (8.5)	17 (70.8)	7 (29.2)		17 (70.8)	7 (29.2)	
Tumor size				0.282			0.001
<5 cm	111 (39.2)	76 (68.5)	35 (31.5)		90 (81.1)	21 (18.9)	
≥5 cm	172 (60.8)	107 (62.2)	65 (37.8)		106 (61.6)	66 (38.4)	
Lymph node status				0.791			0.488
Negative	99 (35.0)	63 (63.6)	36 (36.4)		66 (66.7)	33 (33.3)	
Positive	184 (65.0)	120 (65.2)	64 (34.8)		130 (70.7)	54 (29.3)	
Dissected lymph node counts				0.399			0.292
<28	166 (58.7)	102 (61.4)	62 (37.3)		119 (71.7)	47 (28.3)	
≥28	117 (41.3)	79 (67.5)	38 (32.5)		77 (65.8)	40 (34.2)	
Nervous invasion				0.009			0.977
No	215 (76.0)	130 (60.5)	85 (39.5)		149 (69.3)	66 (30.7)	
Yes	68 (24.0)	53 (77.9)	15 (22.1)		47 (69.1)	21 (30.9)	
Lymphovascular invasion				0.486			0.798
No	211 (74.6)	134 (63.5)	77 (36.5)		147 (51.9)	64 (30.3)	
Yes	72 (25.4)	49 (68.1)	23 (31.9)		49 (68.1)	23 (31.9)	
Histological type				0.804			0.13
Adenocarcinoma	181 (64.0)	118 (65.2)	63 (34.8)		131 (72.4)	50 (27.6)	
Poorly cohesive carcinoma	102 (36.0)	65 (63.7)	37 (36.3)		65 (63.7)	37 (36.3)	
Grade				0.241			0.785
Well	68 (24.0)	48 (70.6)	20 (29.4)		48 (70.6)	20 (29.4)	
Moderate or poor	215 (76.0)	135 (62.8)	80 (37.2)		148 (68.8)	67 (31.2)	
Lauren classification				0.758			0.532
Intestinal	138 (48.8)	88 (63.8)	50 (36.2)		98 (71)	40 (29)	
Diffuse or mixed	145 (51.2)	95 (65.5)	50 (34.5)		98 (67.6)	47 (32.4)	
ypT				0.095			0.150
0–2	64 (22.6)	47 (73.4)	17 (26.6)		49 (76.6)	15 (23.4)	
3–4	219 (77.4)	136 (62.1)	83 (37.9)		147 (67.1)	72 (32.9)	
ypN				0.898			0.353
0	99 (35.0)	63 (63.6)	36 (36.4)		66 (66.7)	33 (33.3)	
1	47 (16.6)	29 (61.7)	18 (38.3)		36 (76.6)	11 (23.4)	
2	75 (26.5)	51 (68.0)	24 (32.0)		55 (73.3)	20 (26.7)	
3	62 (21.9)	40 (64.5)	22 (35.5)		39 (62.9)	23 (37.1)	
ypTNM				0.258			0.412
1	50 (17.7)	36 (72.0)	14 (28.0)		37 (74)	13 (26)	
2	71 (25.1)	41 (57.7)	30 (42.3)		45 (63.4)	26 (36.6)	
3	162 (57.2)	106 (65.4)	56 (34.6)		114 (70.4)	48 (29.6)	
CAP-TRG				0.015			0.050
0–1	88 (31.1)	66 (75.0)	22 (25.0)		68 (77.3)	20 (22.7)	
2–3	195 (68.9)	117 (60)	78 (40)		128 (65.6)	67 (34.4)	
Postoperative chemotherapy				0.609			0.971
No	29 (10.2)	20 (7.1)	9 (3.2)		20 (7.1)	9 (3.2)	
Yes	254 (89.8)	163 (57.6)	91 (35.8)		176 (69.3)	78 (30.7)	

TRG, tumor regression grade; UGEJ, upper third and gastroesophageal junction; cTNM, clinical TNM stage; ypTNM, post-neoadjuvant pathologic stage.

### Relations between NLR, PLR, and TRG

For a more systematic analysis of TRG, we combined NLR and PLR to form four patient subsets, as shown in **Table 3**. The number of cases of the four NLR/PLR groups (low/low, low/high, high/low, and high/high) were, respectively, 145, 38, 51, and 49. In the univariate analysis, both NLR and PLR correlated significantly with TRG (*p* = 0.044), with the TRG of subset NLR^high^/PLR^high^ being significantly worse. Clinical TNM stage (OR = 1.976; *p* = 0.011), tumor size (OR = 3.577; *p* < 0.001), lymph node status (OR = 4.905; *p* < 0.001), nervous invasion (OR = 5.268; *p* < 0.001), lymphovascular invasion (OR = 3.636; *p* < 0.001), tissue staging (OR = 2.691; *p* = 0.001), degree of tumor differentiation (OR = 6.498; *p* < 0.001), Lauren classification (OR = 2.249; *p* = 0.002), ypTNM stage (OR = 76.765; *p* < 0.001), and NLR/PLR (OR = 3.457; *p* = 0.044) were all significantly associated with TRG. In the multivariate analysis (**Table 4**), the NLR^high^/PLR^high^ subset (OR = 6.876; *p* = 0.028) exhibited the worse TRG although the degree of tumor differentiation (OR = 2.874; *p* = 0.037) also correlated with TRG. These results confirmed that NLR and PLR in combination are independently predictive of TRG. TRG was the poorest in the NLR^high^/PLR^high^ patient subset.

**Table 3:** Univariate analysis of CAP-TRG.

Variable	Odds ratio (95% CI)	*p*-value
Age (≥65)	1.110 (0.612, 2.014)	0.731
Gender (female)	0.948 (0.534, 1.686)	0.857
cTNM (3–4)	1.976 (1.171, 3.336)	0.011
Location		0.070
Middle third	1	
UGEJ	2.667 (1.060, 6.711)	0.037
Lower third	1.934 (1.036, 3.682)	0.038
Diffuse	3.145 (1.022, 9.675)	0.046
Tumor size (≥5 cm)	3.577 (2.114, 6.052)	<0.001
Lymph node status (positive)	4.905 (2.859, 8.416)	<0.001
Dissected lymph node counts (>28)	1.099 (0.658, 1.835)	0.719
Nervous invasion (yes)	5.268 (2.298, 12.073)	<0.001
Lymphovascular invasion (yes)	3.636 (1.763, 7.5)	<0.001
Histological type (poorly cohesive carcinoma)	2.691 (1.504, 4.815)	0.001
Grade (moderate or poor)	6.498 (3.593, 11.750)	<0.001
Lauren classification (diffuse or mixed)	2.249 (1.342, 3.770)	0.002
ypT (3–4)	34.444 (15.538, 76.357)	<0.001
ypN		<0.001
0	1	
1	1.856 (0.914, 3.770)	0.087
2	4.609 (2.312, 9.189)	<0.001
3	34.565 (8.002, 149.303)	<0.001
ypTNM		<0.001
1	1	<0.001
2	15.577 (5.492, 44.180)	<0.001
3	76.765 (26.817, 219.745)	<0.001
Adjuvant therapy (yes)	1.188 (0.528, 2.672)	0.678
NLR/PLR		0.044
Low/low	1	
Low/high	1.108 (0.523, 2.347)	0.789
High/low	1.383 (0.693, 2.758)	0.358
High/high	3.457 (1.45, 8.239)	0.050

TRG, tumor regression grade; UGEJ, upper third and gastroesophageal junction; cTNM, clinical TNM stage; ypTNM, post-neoadjuvant pathologic stage.

**Table 4:** Multivariate analysis of CAP-TRG.

Variable	Odds ratio (95% CI)	*p*-value
cTNM (3–4)	1.225 (0.479, 3.131)	0.671
Tumor size (≥5 cm)	0.837 (0.361, 1.936)	0.677
Lymph node status (positive)	2.955 (0.263, 33.232)	0.380
Nervous invasion (yes)	1.908 (0.689, 5.281)	0.213
Lymphovascular invasion (yes)	2.428 (0.859, 6.865)	0.094
Histological type (poorly cohesive carcinoma)	1.394 (0.569, 3.415)	0.467
Grade (moderate or poor)	2.874 (1.069, 7.728)	0.037
Lauren classification (diffuse or mixed)	0.932 (0.384, 2.264)	0.877
ypT (3–4)	7.033 (0.830, 59.620)	0.074
ypN		0.062
0	1	
1	0.357 (0.069, 1.860)	0.221
2	0.848 (0.126, 5.713)	0.865
3	2.955 (0.263, 33.232)	0.380
ypTNM		0.474
1	1	
2	3.283 (0.355, 30.391)	0.295
3	8.733 (0.266, 286.647)	0.224
NLR/PLR		0.028
Low/low	1	
Low/high	0.837 (0.265, 2.637)	0.761
High/low	1.329 (0.497, 3.553)	0.571
High/high	6.876 (1.857, 25.454)	0.040

TRG, tumor regression grade; UGEJ, upper third and gastroesophageal junction; cTNM, clinical TNM stage; ypTNM, post-neoadjuvant pathologic stage.

### Relations between NLR, PLR, and prognosis

**Figure 3:**
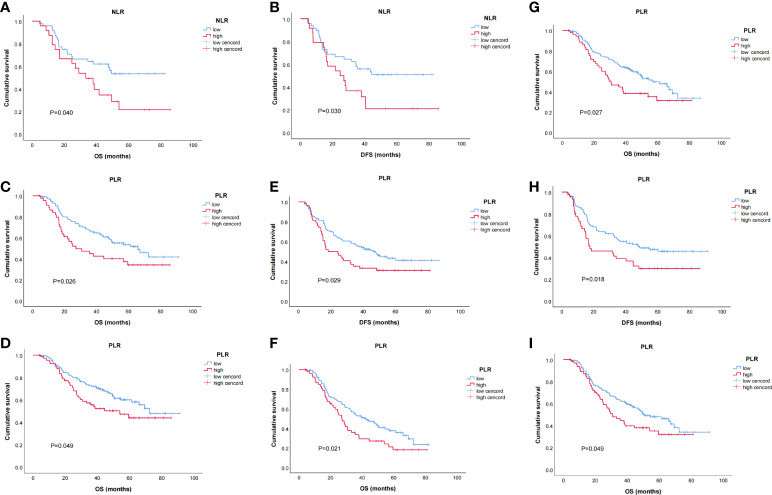
Kaplan–Meier survival curves of stratified baseline characteristics. **(A)** OS for neutrophil-to-lymphocyte ratio (NLR) in female patients (*p* = 0.040); **(B)** disease-free survival (DFS) for NLR in female patients (*p* = 0.030);**(C)** OS for PLR in patients with clinical TNM 3 stage (*p* = 0.027); **(D)** OS for PLR in patients with dissected lymph node counts < 28 (*p* = 0.026); **(E)** DFS for PLR in patients with clinical TNM 3 stage (*p* = 0.029); **(F)** DFS for PLR in patients with dissected lymph node counts < 28 (*p* = 0.018); **(G)** OS for platelet-to-lymphocyte ratio (PLR) in patients age < 65 (*p* = 0.049); **(H)** OS for PLR in patients with positive lymph nodes (*p* = 0.021); **(I)** OS for PLR in patients with moderate or poorly differentiated tumors (*p* = 0.049).

The relations between NLR, PLR, and prognosis were analyzed according to clinicopathological factors, as shown in **Table 5**. In female patients, low NLR patients had significantly better OS (*p* = 0.04) and DFS (*p* = 0.03) than high NLR patients (**Figures 3A, B**). In patients with clinical TNM stage 3 disease and dissected nodal counts <28, OS (*p* = 0.027, 0.026) and DFS (*p* = 0.029, 0.018) were also significantly better in the low PLR (vs. high PLR) subset (**Figures 3C–F**). For patients <65 years old, those with positive lymph nodes, or those with moderate or poor tumor differentiation, OS was significantly better in the low PLR (vs. high PLR) subset (*p* = 0.049, 0.021, 0.049, respectively)(**Figures 3G–I**).

**Table 5:** Stratified survival analysis of baseline characteristics.

Variable	NLR (*p*-value)		PLR (*p*-value)	
	OS	DFS	OS	DFS
Age				
<65	0.160	0.135	**0.049**	0.085
≥65	0.645	0.969	0.793	0.615
Gender				
Male	0.868	0.833	0.655	0.679
Female	**0.040**	**0.030**	0.072	0.143
cTNM				
Stage 2	0.569	0.789	0.705	0.520
Stages 3–4	0.609	0.289	**0.027**	**0.029**
Tumor size				
<5 cm	0.706	0.669	0.518	0.600
≥5 cm	0.625	0.432	0.495	0.599
Lymph node status				
Negative	0.462	0.216	0.934	0.943
Positive	0.282	0.318	**0.021**	0.054
Dissected lymph node counts				
<28	0.748	0.256	**0.026**	**0.018**
≥28	0.298	0.719	0.934	0.616
Nervous invasion				
No	0.260	0.167	0.361	0.463
Yes	0.509	0.419	0.226	0.270
Lymphovascular invasion				
No	0.301	0.234	0.178	0.389
Yes	0.937	0.646	0.756	0.541
Histological type				
Adenocarcinoma	0.720	0.535	0.602	0.722
Poorly cohesive carcinoma	0.321	0.305	0.185	0.255
Grade				
Well	0.502	0.247	0.498	0.277
Moderate or poor	0.306	0.124	**0.049**	0.057
Lauren classification				
Intestinal	0.367	0.318	0.723	0.895
Diffuse or mixed	0.662	0.484	0.153	0.116
ypT				
0–2	0.854	0.722	0.561	0.490
3–4	0.629	0.373	0.390	0.588
ypTNM				
1	0.822	0.647	0.100	0.070
2	0.404	0.153	0.529	0.391
3	0.444	0.469	0.056	0.108

TRG, tumor regression grade; UGEJ, upper third and gastroesophageal junction; cTNM, clinical TNM stage; ypTNM, post-neoadjuvant pathologic stage. The numbers in bold indicate that these P-values less than 0.05 are statistically significant.

## Discussion

Herein, we retrospectively evaluated the data from 283 patients with locally advanced GC, each subjected to NCT and subsequent surgical resection. We then explored the relations of NLR and PLR with clinicopathological factors, TRG, and prognosis. Our efforts indicate that NLR and PLR together are independently predictive of TRG. They may predict OS and DFS as well.

Inflammation plays a crucial role in the development of malignancy. Circulating neutrophil levels in humans usually reflect the body’s systemic inflammatory response. Lymphocytic immunocytes are important components of human tumor-specific immune responses and active participants in tumor eradication. The NLR may indirectly measure the inflammatory/immune status in patients with tumors, gauging the balance between pro- and antitumor inflammatory responses and, thus, inferring the biologic behavior and prognosis of cancers. A higher NLR indicates fewer lymphocytes relative to neutrophils. It signals a disruption in equilibrium, favoring tumor promotion and a poorer prognosis.

Tumor response is one of the most important prognostic factors in patients who undergo NCT for locally advanced GC. Because clinicopathological data obtained prior to treatment are tenuous in predicting tumor response, we focused instead on preoperative NLR and PLR, analyzing their relations to first-line chemotherapeutic outcomes (*via* SOX, XELOX) in this setting. Multivariate analysis of our data showed the close links of NLR and PLR with chemotherapeutic efficacy. The lower their values proved, the better the chemotherapeutic efficacy. Hence, NLR and PLR together may be useful as independent predictors of NCT efficacy in patients with locally advanced GC.

Certain studies have affirmed the validity of a systemic immune-inflammation index in predicting pathological response and patient prognosis after NCT for breast cancer (19, 20). Powell et al. (21) have also reported an association between low pretreatment NLRs and good pathological responses in patients with esophageal cancer, and Caputo et al. (22) have documented both poorer TRG and increased incidence of postoperative complications in patients with rectal cancers and high preoperative NLRs. Our results are consistent with those above. It is feasible that fewer lymphocyte numbers equate with reduced bodily antitumor immunity, hindering the ability to recognize and respond to tumor antigenic mutations. This in turn encourages the escape of tumor cells, creating an environment favorable for their proliferation and metastasis and ultimately acting to confer drug resistance. Under the actions of platelets, cancer cells are inclined to upregulate anti-apoptotic genes, downregulate pro-apoptotic genes, and increase the expression levels of cell-cycle proteins, DNA repair proteins, and mitogen-activated protein kinases (MAPKs). Secretome analysis following platelet–cancer cell interactions has detected the enhanced release of RANTES (regulated upon activation, normal T-cell expressed and secreted), thrombospondin-1, transforming growth factor-β, and clusterin chemokines. These substances bolster the drug resistance of cancer cells (23).

We also evaluated NLR and PLR in relation to prognosis. In female patients, OS and DFS were significantly better in the low NLR (*vs*. high-NLR) patient subset. NLR has been shown by others to correlate with chemotherapeutic responses in several types of cancer. Powell et al. (21) noted a relation between low pretreatment NLR and good OS after NCT for esophageal cancer, and Chua et al. (12) identified NLR as an independent predictor of OS in patients with advanced colorectal cancer. However, the precise mechanisms behind such revelations are not entirely clear. One explanation may be that vascular endothelial growth factor (VEGF) is primarily derived from neutrophils, and VEGF overexpression is among the acknowledged conditions required to promote tumor angiogenesis and distant metastasis. Neutrophilia may also serve to promote oxygen-free radical release, inflict cellular DNA damage, inactivate tumor suppressor genes, activate oncogenes, and further tumor development (24, 25). According to our data, the higher the NLR proved, the worse the chemotherapeutic response and the worse the prognosis.

PLR is a systemic immune-inflammatory index shown in recent years to predict malignant tumor prognosis. In the present study, PLR was predictive of survival in patients <65 years old and in those with clinical TNM stage 3 disease, dissected nodal counts <28, lymph node positivity, or moderate/poor tumor differentiation. The higher the PLR, the worse the prognosis appeared. Our observations are aligned with the results of other studies (26–28), although the specific mechanisms have yet to be elucidated. However, malignant cells trigger the production of myeloid-stimulating cytokines (i.e., interleukin 6, tumor necrosis factor-α, and growth factors) that contribute to thrombocytosis. Activated platelets may then promote tumor proliferation, angiogenesis, metastasis, and cancer-associated thrombosis through the release of growth factors, such as platelet-derived growth factor, platelet factor 4, and platelet-reactive protein (29). Another consideration is the synergism between platelets and neutrophils. Neutrophil recruitment may depend on the presence of platelets, which adhere to the endothelium, bringing endothelial cells and neutrophils in contact (30). Platelets may similarly supply chemokines to attract neutrophils in large arteries (31).

This particular investigation had some limitations, one being the potential for selection bias inherent in a single-center retrospective review. The small sample size may have contributed as well to the large OR values cited. Another issue is that the cutpoints we determined differed from those referenced elsewhere, thus prohibiting comparisons with prior results. Still, the samples we analyzed were consistently from patients with locally advanced gastric adenocarcinomas, and the cutpoints calculated were of high credibility, based on the ROC curves and the Youden index.

The strengths of this study include our robust follow-up data. Follow-up durations were reasonable, and mortality causes/dates were accurately recorded. Furthermore, a National Health Service Laboratory using standardized methods conducted all serum analyses and tissue examinations, ensuring their reliability and reproducibility.

## Conclusion

NLR and PLR may predict pathological responses and prognoses after NCT and surgery in patients with locally advanced GC. Screening for patients with high NLR and PLR values may allow them to benefit upfront from alternatives to NCT. The clinical application of NLR and PLR is currently limited by inconsistent, dichotomous thresholds.

## Data availability statement

The raw data supporting the conclusions of this article will be made available by the authors, without undue reservation.

## Ethics statement

This study was reviewed and approved by The ethics committee of Liaoning Cancer Hospital and Institute. The patients/participants provided their written informed consent to participate in this study.

## Author contributions

WW analyzed the data and drafted the manuscript. SS and YT reviewed and revised the manuscript. YLT, ZS, FS, and CJ assisted in data collection and analyzed the data. YZ and JZ supervised the study and provided critical revision of the manuscript. All authors contributed to the article and approved the submitted version.

## Funding

This study was supported by grants from the Key Research and Development Program of Liaoning Province (2020JH2/10300043), the Natural Science Foundation of Liaoning Province (2020-ZLLH-45), and the Shenyang High-Level Innovative Talents Program (RC190447).

## Conflict of interest

The authors declare that the research was conducted in the absence of any commercial or financial relationships that could be construed as a potential conflict of interest.

The reviewer BJ declared a shared parent affiliation with the authors to the handling editor at the time of review.

## Publisher’s note

All claims expressed in this article are solely those of the authors and do not necessarily represent those of their affiliated organizations, or those of the publisher, the editors and the reviewers. Any product that may be evaluated in this article, or claim that may be made by its manufacturer, is not guaranteed or endorsed by the publisher.

## Abbreviations

NLR, neutrophil-to-lymphocyte ratio; PLR, platelet-to-lymphocyte ratio; GC, gastric cancer; TRG, tumor regression grade; NCT, neoadjuvant chemotherapy; ROC, receiver operating characteristic; OS, overall survival; DFS, disease-free survival; AJCC, American Joint Committee on Cancer; ANC, absolute value of neutrophil count; ALC, absolute value of lymphocyte count; PLT, platelet count; UGEJ, upper third and gastroesophageal junction; SOX, S-1 plus oxaliplatin; XELOX, oxaliplatin and capecitabine; CAP-TRG, College of American Pathologists TRG system; VEGF, vascular endothelial growth factor.
